# Stage, age, and EBV status impact outcomes of plasmablastic lymphoma patients: a clinicopathologic analysis of 61 patients

**DOI:** 10.1186/s13045-015-0163-z

**Published:** 2015-06-10

**Authors:** Sanam Loghavi, Khaled Alayed, Tariq N. Aladily, Zhuang Zuo, Siok-Bian Ng, Guilin Tang, Shimin Hu, C. Cameron Yin, Roberto N. Miranda, L. Jeffrey Medeiros, Joseph D. Khoury

**Affiliations:** Department of Hematopathology, The University of Texas, M.D. Anderson Cancer Center, 1515 Holcombe Boulevard, MS-072, Houston, TX 77030 USA; Department of Pathology, King Saud University, Riyadh, Saudi Arabia; Department of Pathology and Laboratory Medicine, The University of Jordan, Amman, Jordan; Department of Pathology, National University of Singapore and Cancer Science Institute, Singapore, Singapore

**Keywords:** Plasmablastic lymphoma, EBV, HIV, Immunodeficiency, Immunocompetent, MYC

## Abstract

**Background:**

Plasmablastic lymphoma (PBL) is a rare aggressive neoplasm with lymphoid and plasmacytic differentiation that is commonly associated with immunodeficiency and an unfavorable prognosis. Clinicopathologic features have been largely derived from cases reports and small series with limited outcome analyses.

**Patients and methods:**

The demographic, clinicopathologic features, and clinical outcomes of a cohort of 61 patients with PBL were reviewed and analyzed.

**Results:**

Patients had a median age of 49 years (range 21–83 years) and most (49/61; 80 %) were men. Human immunodeficiency virus (HIV) status was available for 50 patients: 20 were HIV-positive and 30 HIV-negative. Twenty-three patients were immunocompetent. Abdominal/gastrointestinal complaints were the most common presenting symptoms, reported in 14 of 47 (30 %) of patients. At presentation, 24 of 43 (56 %) patients had stage III or IV disease. Epstein-Barr virus (EBV) was detected in 40 of 57 (70 %) cases. *MYC* rearrangement was identified in 10/15 (67 %) cases assessed, and MYC overexpression was seen in all cases assessed regardless of *MYC* rearrangement status. HIV-positive patients were significantly younger than those who were HIV-negative (median 42 vs. 58 years; *p* = 0.006). HIV-positive patients were also significantly more likely to have EBV-positive disease compared with HIV-negative patients (19/19, 100 % vs. 15/29, 52 %; *p* = 0.002). Patients who received CHOP chemotherapy tended to have better overall survival (OS) compared with those who received hyperfractionated cyclophosphamide, vincristine, doxorubicin, and dexamethasone (hyper-CVAD) (*p* = 0.078). HIV status had no impact on OS. Patients with EBV-positive PBL had a better event-free survival (EFS) (*p* = 0.047) but not OS (*p* = 0.306). Notably, OS was adversely impacted by age ≥50 years (*p* = 0.013), stage III or IV disease (*p* = <0.001), and lymph node involvement (*p* = 0.008).

**Conclusions:**

The most significant prognostic parameters in patients with PBL are age, stage, and, to a lesser extent, EBV status. In this study, two-thirds of PBL cases assessed were associated with *MYC* rearrangement and all showed MYC overexpression.

**Electronic supplementary material:**

The online version of this article (doi:10.1186/s13045-015-0163-z) contains supplementary material, which is available to authorized users.

## Introduction

Plasmablastic lymphoma (PBL) is a rare type of non-Hodgkin lymphoma in which the neoplastic cells are postulated to arise from plasmablasts, defined as short-lived B cells that have switched their transcriptional phenotype to a plasma cell gene expression program [1]. Under physiologic conditions, sensitized memory B cells activated by repeated exposure to antigen can differentiate within approximately 7 days into plasmablasts capable of immunoglobulin heavy chain (IgH) class switching [2]. While the canonical immunophenotype of such plasmablasts is CD19^low^/CD20^−^/CD38^high^/CD138^−/+^, an intermediate CD20^+^ pre-plasmablast phase has been identified [2, 3]. Notably, infection by human immunodeficiency virus (HIV) and Epstein-Barr virus (EBV), or both, are known to cause an unusual surge in plasmablast levels in the blood and lymph nodes.

Although PBL commonly occurs in HIV-positive individuals, it may also arise in association with other immunodeficiency or immunocompromised states such as organ transplantation, autoimmune diseases, and older age, as well as in immunocompetent individuals. Among HIV patients, PBL constitutes an acquired immunodeficiency syndrome (AIDS)-defining condition. Currently, PBL is defined as a high-grade lymphoma comprised of a diffuse proliferation of large neoplastic cells that resemble immunoblasts or plasmablasts expressing an immunophenotype resembling that of plasma cells [1, 4].

The scope of clinicopathologic features and outcomes of patients with PBL remains poorly characterized, in large part due to the rarity of this entity whose features have been largely pieced together through case reports and small case series. In a recent study in which Morscio et al. assessed 28 cases of PBL from their institution and performed a literature review, they suggested a framework for categorizing PBL patients into three broad categories: (1) HIV-positive, (2) immunocompetent, and (3) post-transplantation [5]. In this study, we build on this proposed framework and analyze the clinicopathologic features, therapeutic approaches, and clinical outcomes of the largest cohort of PBL patients reported to date.

## Results

### Clinical features

Most patients were men (80 %) with a median age of 49 years (range 21–83 years). The most common presenting symptoms included abdominal/gastrointestinal complaints (e.g., diarrhea, hematochezia, pain) (14/47; 30 %), localized mass or swelling (12/47; 26 %), and oral/nasal symptoms (e.g., ulcer, epistaxis, rhinorrhea, sinusitis) (8/47; 17 %). Lymph node involvement was identified in 34 of 49 (69 %) patients. Extranodal disease was relatively common, with the oral/nasal cavity (21/46; 46 %) and gastrointestinal tract (12/60; 20 %) being the most commonly involved extranodal sites. Among patients with available staging information, 19/43 (44 %) presented with Ann Arbor stage I/II disease and 24/43 (56 %) presented with stage III/IV disease.

Patients were divided into four clinical categories: HIV-positive (PBL-HIV) (*n* = 20), post-transplant (PBL-PT) (*n* = 3), autoimmune disease (PBL-AD) (*n* = 4), and immunocompetent (PBL-IC) (*n* = 23) (Table 1). Excluded from these categories (and thus analyses of clinical features) are 11 patients for whom HIV status was not available. Patients in the PBL-HIV category were significantly younger (median 41.7 vs. 57.8 years; *p* = 0.006) than patients in other categories. All PBL-HIV cases assessed were EBV-positive compared with 52 % among cases arising in patients within other categories (19/19 vs. 15/29; *p* = 0.002). Receipt of combined antiretroviral therapy (cART) was documented for 17 HIV-positive patients; no data regarding cART therapy was available for the remaining 3 patients.

**Table 1:** Clinical features of plasmablastic lymphoma patients in this study group

	Total	PBL-HIV	PBL-PT	PBL-AD	PBL-IC	*p* value	*p* value	*p* value
	*N* (%)	*N* (%)	*N* (%)	*N* (%)	*N* (%)	(HIV vs. IC)	(HIV vs. other)	(IC vs. other)
Patients^a^	61 (100)	20 (33)	3 (5)	4 (7)	23 (38)			
Age						0.026	0.006	0.141
Median (years)	49.2	41.7	66	52	55.7			
Range (years)	21–83	30–63	29.1–73.0	38–76	26–81			
Sex						0.075	0.092	0.073
Male	49 (80)	19 (95)	2 (67)	3 (75)	18 (78)			
Female	12 (20)	1 (5)	1 (33)	1 (25)	5 (22)			
Race						0.362	0.275	0.203
Caucasian	33 (54)	11 (55)	3 (100)	1 (25)	12 (52)			
Hispanic	9 (15)	2 (10)		1 (25)	5 (22)			
Black	2 (3)	2 (10)			0			
Asian	7 (12)	2 (10)		1 (25)	3 (13.0)			
Unspecified	10 (16)	3 (15)		1 (25)	3 (13.0)			
Site(s) of involvement^b^						0.469	0.526	0.406
Nasal/oral cavity	21/46 (46)	9 (45)			6 (26)			
Lymph node	34/49 (69)	14 (70)	1/1 (100)	1/2 (50)	13/21 (62)			
GI tract	12 (20)	3 (15)	1(33)	3 (75)	3 (13.0)			
Bone	7 (12)	4 (20)			3 (13.0)			
Bone marrow	8/44 (18)	1/18 (6)	1 (33)	1/3 (33)	4/18 (22)			
Abdominal/pelvic cavity	6 (10)	2 (10)	1 (33)	1 (25)	2 (9)			
Body fluid	6 (10)	1 (5)	1 (33)		2 (9)			
Liver	2 (3)	1 (5)		1 (25)				
Skin	2 (3)							
Soft tissue	2 (3)				2 (9)			
Retroperitoneum	2 (3)	1 (5)	1 (33)					
Mediastinum	2 (3)				1 (4)			
Tonsil	1 (2)				1 (4)			
Testis	1 (2)				1 (4)			
Penis	1 (2)				1 (4)			
Spleen	1 (2)	1 (5)						
Gynecologic organs	1 (2)	1 (5)						
Breast	1 (2)				1 (4)			
Bladder	1 (2)				1 (4)			
Kidney	1 (2)	1 (5)						
NA	1 (2)				0			
Ann Arbor stage at diagnosis						0.979	0.577	0.619
I + II	19/43 (44)	4 (20)		1 (25)	6 (26)			
III + IV	24/43 (56)	3 (15)		1 (25)	4 (17)			
Chemotherapy						0.495	0.431	0.889
H-CVAD	16/42 (38)	5 (25)	1 (33)	2 (50)	8 (50)			
CHOP	8/42 (19)	4 (20)	1 (33)		3 (13)			
EPOCH	7/42 (17)	4 (20)	1 (33)	1 (25)	1 (4)			
Other	11/42 (26)	1 (5)		1 (25)	6 (14)			
Radiation therapy	18/41 (44)	5/15 (33)	1 (33)	0/3	11/18 (61)			
Stem cell transplant	6/42 (14)	1/15 (7)	1 (33)	0/3	4/19 (21)			
Survival (months)						0.198	0.500	0.184
Median	7	14	35	2	17			
Range	0.3–156	0.3–120	9–156	1–7	0.2–150			
Status at last follow-up								
Dead	43 (71)	15 (75)	3 (100)	2 (50)	6 (26)			
Alive	18 (30)	5 (25)	0	2 (50)	17 (74)			

*PBL* plasmablastic lymphoma, *HIV* human immunodeficiency virus, *PT* post-transplant, *AD* autoimmune disease, *IC* immunocompetent, *EPOCH* etoposide, prednisone, vincristine, cyclophosphamide, doxorubicin, *CHOP* cyclophosphamide, doxorubicin, vincristine, prednisone, *H*-*CVAD* hyperfractionated cyclophosphamide, vincristine, doxorubicin, dexamethasone, methotrexate, cytarabine, *NA* not available

^a^HIV status for 11 patients was unknown

^b^Some patients had more than one site of involvement; therefore, the cumulative data may exceed 100 %

Patients in the PBL-PT category included one patient (case 2) who had received allogeneic SCT for accelerated chronic lymphocytic leukemia/small lymphocytic lymphoma (CLL/SLL) 7 years prior to developing PBL and two patients (cases 1 and 15) who had received liver transplants. Patients in the PBL-AD category included one patient with rheumatoid arthritis, one with ulcerative colitis, one with Crohn disease, and one with Sjögren syndrome. Patients in the PBL-IC group had no apparent evidence of immunodeficiency and were by default regarded as immunocompetent. Since no agreed upon cutoff exists for age-related decline in immunocompetence, patients were not grouped a priori on the basis of age. Cutoffs of 50 and 60 years were assessed for prognostic significance, and both were found to be associated with overall survival (OS) (see below).

**Fig. 1:**
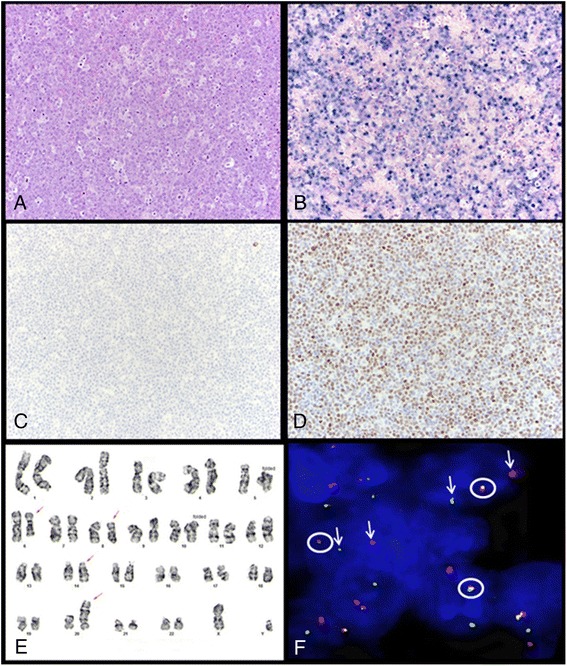
Representative case of plasmablastic lymphoma. **a** Neoplastic cells have plasmablastic morphology, with a prominent nucleolus and moderate amount of cytoplasm. Mitotic figures and tingible-body macrophages are abundant and impart a “starry-sky” pattern (H&E, ×200). **b** The neoplastic cells are diffusely positive for EBV-encoded RNA (EBER) by colorimetric in situ hybridization (×200). **c** CD20 expression is absent. This case was negative for CD19 and positive for CD38 by flow cytometry (data not shown) (×200). **d** MYC overexpression is positive by immunohistochemistry (×200). **e** Karyotype of case 30 (nasopharyngeal mass): 46, XY, del(6)(q23q29),t(8;14)(q24;q32), add(20)(p13). **f** Fluorescence in situ hybridization using a dual-color break-apart probe specific for the *MYC* locus on formalin-fixed paraffin-embedded tissue (case 30) showing split signals in ~80 % of nuclei (*circle*: fusion signal; *arrow*: split signal)

Five patients in our study group had a history of lymphoid malignancy. One patient with CLL/SLL was mentioned above. Two patients (cases 30 and 48) had a history of diffuse large B cell lymphoma (DLBCL), and one patient (case 29) had a history of Burkitt lymphoma. One of the patients with DLBCL (case 30) and the patient with Burkitt lymphoma were HIV-positive. Interestingly, the former patient (case 30) developed PBL with t(8;14)(q24.1;q32) and *MYC*/*IGH* rearrangement 8 years following therapy for DLBCL (Fig. 1e, f). The second patient with DLBCL (case 48) had a composite lymphoma consisting of a conventional DLBCL and PBL, each component with typical morphology and immunophenotype. The fifth patient (patient 34) had a remote history of lymphoma according to the clinical notes; the original lymphoma was not available to us for review.

### Histopathologic features

An incisional/excisional biopsy specimen was obtained in 41 (67 %) patients, needle biopsy in 18 (30 %) patients, fine needle aspiration biopsy in 1 (2 %) patient, and bone marrow aspiration and biopsy in 1 (2 %) patient. Morphologic evaluation was limited in a small subset of cases due to limited material, extensive necrosis, and/or crush artifact. All cases had a diffuse growth pattern. A “starry-sky” pattern was seen in 22 cases and necrosis in 14 cases. The neoplastic cells exhibited exclusively plasmablastic morphology in 52 cases, whereas in 4 cases they consisted of an admixture of plasmablasts and plasmacytic cells. Large, pleomorphic multinucleated cells were identified in six cases.

### Immunophenotyping and colorimetric in situ hybridization results

**Fig. 2:**
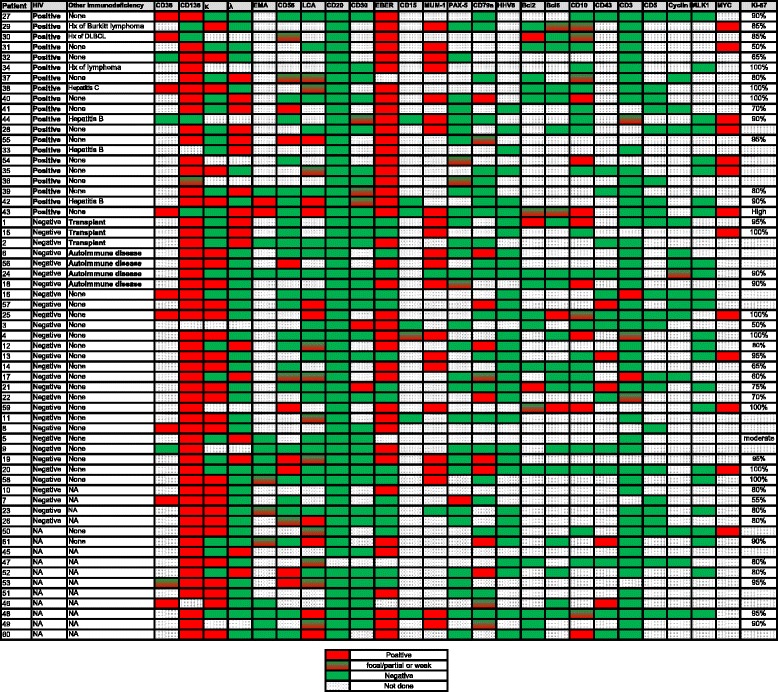
Schematic representation of the immunophenotypic features of plasmablastic lymphoma cases in this study

Colorimetric in situ hybridization for EBV-encoded RNA (EBER) was positive in 40/57 (70 %) cases. Among cases assessed for immunoglobulin light chain expression, 45/52 (87 %) expressed cytoplasmic light chain: 28 kappa and 17 lambda. The neoplastic cells were also positive for the following antigens: CD138 (54/58; 93 %); MUM1 (22/24; 92 %); CD38 (10/13; 77 %); CD45 (20/40; 50 %); CD79a (13/35; 37 %); CD10 (13/32; 41 %); CD56 (12/37; 32 %); BCL2 (5/20; 25 %); CD43 (5/19; 26 %); epithelial membrane antigen (EMA) (5/16; 31 %); BCL6 (4/22; 18 %); CD15 (1/7; 14 %); CD30 (5/38; 13 %); PAX5 (4/32; 13 %); and CD3 (5/51; 10%, mostly focal, weak). Two cases were assessed for CD117 and both were positive. Cyclin D1 was focally positive in 1/15 (7 %) cases. All cases assessed were negative for CD20 (*n* = 60), and HHV8 (*n* = 33). Ki-67 was assessed in 43 cases demonstrating a median Ki-67 proliferation index of 90 %. A summary of the immunophenotypic features of PBL in patients within various clinical categories is provided in Table 2 and further illustrated in Fig. 2.

**Table 2:** Summary of immunophenotypic features and MYC rearrangement status

	Total *N* (%)	PBL-HIV *N* (%)	PBL-PT *N* (%)	PBL-AD *N* (%)	PBL-IC N (%)
CD10	13/32 (41)	6/14 (43)	1/2 (50)	1/2 (50)	3/10 (30)
CD20	0/60	0/20	0/3	0/4	0/23
CD45	20/40 (50)	6/11 (54)	0/1	0/2	7/18 (39)
CD56	12/37 (32)	4/11 (36)	0/3	1/3 (33)	5/15 (33)
CD79a	13/35 (37)	2/11 (18)	0/1	1/3 (33)	6/13 (46)
CD138	54/58 (93)	15/19	3/3 (100)	4/4 (100)	22/23 (96)
MUM1	22/24 (92)	8/8 (100)	2/2 (100)	3/4 (75)	7/8 (88)
MYC	13/13 (100)	7/7 (100)	1/1 (100)	0/0 (0)	4/4 (100)
Ki-67 (% median)	90	87.5	97.5	90	80
EBER (ISH)	40/57 (70)	19/19 (100)^*^	1/3 (33)	2/4 (50)	12/22 (55)
*MYC* rearrangement (FISH)	10/15 (67)	5/7 (71)^**^	1/1 (100)	NA	3/6 (50)

*PBL* plasmablastic lymphoma, *HIV* human immunodeficiency virus, *PT* post-transplant, *AD* autoimmune disease, *IC* immunocompetent, *EBER* Epstein- Barr virus-encoded RNA, *ISH in situ* hybridization; *FISH* fluorescence *in situ* hybridization, *NA* not available

*p = <0.001 (PBL-HIV vs. others); **p = 0.323 (PBL-HIV vs. others)

### Conventional cytogenetics and fluorescence in situ hybridization results

Conventional cytogenetic analysis results were available for 13 cases, of which 5 had clonal chromosomal abnormalities (Additional file 1: Table S1), 7 had a diploid karyotype, and 1 had abnormal non-clonal metaphases with numerical and structural changes. Abnormalities of chromosome 8 were identified in 4 cases including 2 cases with t(8;14)(q24;q32).

Fluorescence in situ hybridization (FISH) using probes specific for the *MYC* locus was performed on 15 cases, of which 10 (67 %) were positive for *MYC* gene rearrangement. Notably, there was no significant association between *MYC* rearrangement and clinical categories. We performed immunohistochemistry to assess MYC protein expression in a subset of cases with (*n* = 10) and without (*n* = 3) available *MYC* status by FISH and/or conventional karyotyping for which tissue was available. All cases assessed showed of MYC overexpression regardless of *MYC* rearrangement status. However, the extent (median 90 % positive nuclei; range 60–100 % vs. median 75 % positive nuclei; range 60–100 %) and intensity (3+ vs. 2+) of MYC overexpression were more pronounced in cases with *MYC* rearrangement (*n* = 6) compared with those without *MYC* rearrangement (*n* = 4).

Horn et al. recently reported that CD10 expression is more commonly seen in diffuse large B cell lymphomas with immunoblastic morphology and *MYC* rearrangements compared with cases with intact *MYC* [6]. Accordingly, we asked whether such a correlation might hold true for PBL, particularly in view of the seemingly consistent presence of MYC overexpression in this disease. Interestingly, there was no significant difference in CD10 expression between cases with and without *MYC* rearrangement in the small group of PBL cases we were able to assess (3/7; 43 % vs. 1/4; 25 %, respectively; *p* = 1.000).

### Treatment

Treatment details were available for 42 (69 %) patients, of whom 3 did not receive any form of therapy for PBL. Six patients had a history of having received therapy for PBL, but further details were not available. Treatment modalities for the 33 patients who were treated and for whom therapy details were available were as follows: 16 (48 %) patients received chemotherapy alone, 17 (52 %) received chemotherapy and radiation therapy, and 1 patient (3 %) received radiation therapy alone. Subsequently, 6 (18 %) patients underwent autologous SCT (4 following chemotherapy and radiation therapy; 2 following chemotherapy alone). One patient underwent allogeneic SCT 6 months after relapse following autologous SCT.

Chemotherapy regimens used are summarized in Table 1. They included hyperfractionated cyclophosphamide, vincristine, doxorubicin, and dexamethasone (hyper-CVAD) (15/33; 45 %); CHOP (7/33; 21 %); etoposide, vincristine, doxorubicin, cyclophosphamide, and prednisone (EPOCH) (7/33; 21 %); and CHOP followed by hyper-CVAD (1/33; 3 %). Two patients received vincristine and prednisone, and one patient received cyclophosphamide, vincristine, and prednisone (CVP).

### Outcome analysis

The median follow-up duration was 7 months (range, 0.3–156 months). At last follow up, 43 (71 %) of PBL patients were dead and 18 (30 %) were alive. Among patients who received therapy, 19/39 (49 %) achieved complete remission and, with the exception of a single patient who died of a cardiac cause, were alive at last follow up. On the other hand, 14/39 (36 %) patients had persistent disease, and 11 of these were dead at last follow up.

**Fig. 3:**
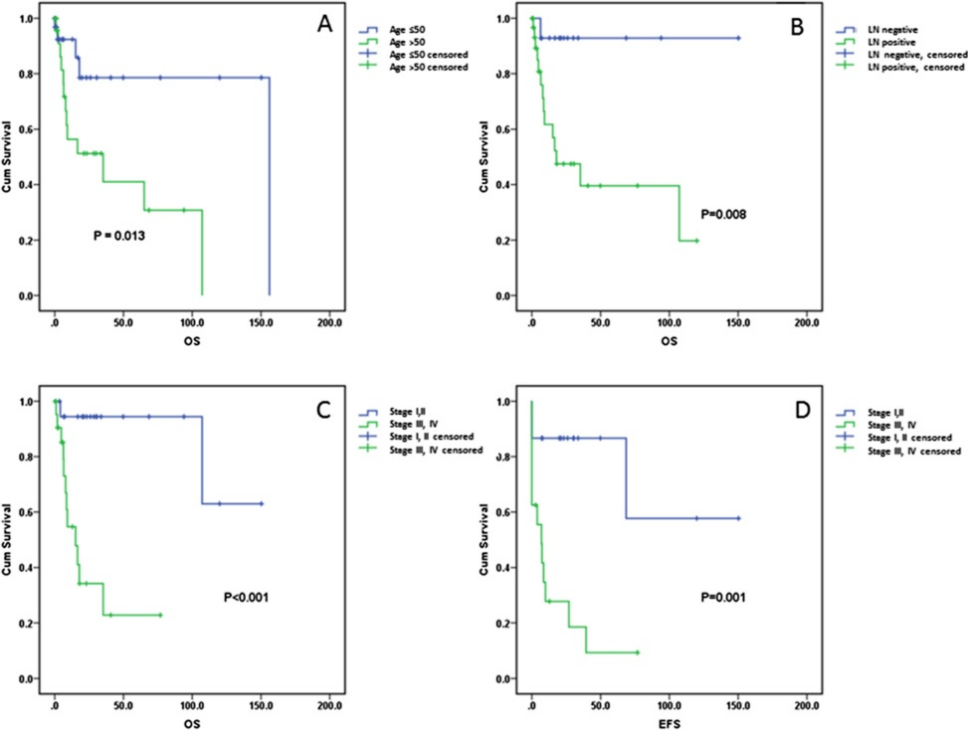
Overall survival of plasmablastic lymphoma patients showing a significant adverse impact of age >50 years (**a**) and lymph node involvement (**b**). Patients who presented with Ann Arbor stage III or IV disease had worse overall (**c**) and event-free survival (**d**) compared to patients who presented with stage I or II

Overall survival (OS) was more favorable for patients <50 years of age compared with those ≥50 years (*p* = 0.013) (Fig. 3a). A similar finding was identified using 60 years as a cutoff (*p* = 0.001). Whereas involvement of visceral organs did not impact OS (*p* = 0.759), lymph node involvement was associated with a significantly worse OS (*p* = 0.008) (Fig. 3b). In addition, patients who presented with stage I/II disease had more favorable OS and event-free survival (EFS) compared to those who presented with stage III/IV disease (*p* = <0.001 and 0.001, respectively) (Fig. 3c, d). Bone marrow involvement was also associated with a worse OS and EFS (*p* = 0.033 and 0.016, respectively). When we compared the OS of patients who received CHOP alone to those who received only hyper-CVAD, the former tended to have a better OS, although the difference did not attain statistical significance (*p* = 0.078). EBV-positive status was associated with a better EFS compared to EBV-negative status for the entire patient cohort (*p* = 0.047) as well as patients <60 years (*p* = 0.015). No association between EBV status and OS was identified (*p* = 0.306). HIV status (*p* = 0.538), autoimmune disease (*p* = 0.235), transplant history (*p* = 0.921), and oral/nasal cavity involvement (*p* = 0.744) had no impact on OS. By multivariate analysis using Cox regression modeling, age was independently associated with OS (HR 1.191; 95 % CI 1.023–1.388; *p* = 0.024). Although advanced stage, EBV status, and HIV status were associated with an elevated hazards ratio, they were not independently associated with OS (Additional file 1: Table S2).

## Discussion

Plasmablastic lymphoma is an uncommon type of non-Hodgkin lymphoma with overlapping features of lymphoma and plasma cell neoplasms [7–9]. Although earlier studies described neoplasms that, in retrospect, were likely examples of PBL [10], it was not until 1997 that PBL was recognized as a distinct entity. At that time, Delecluse et al. described 16 patients who had aggressive B cell lymphoma with plasmacytic differentiation characterized clinically by a predilection for the oral cavity and a high association with both HIV and EBV infection [11]. Many case reports and small case series have since been published, and the concept of PBL has been expanded to include similar tumors in non-oral sites arising in patients with other forms of immunodeficiency (HIV-negative) as well as immunocompetent patients [12, 13].

The high association of PBL with immunosuppression, oral cavity involvement, male gender, EBV infection, and aggressive clinical behavior [14] has been traditionally stressed in the literature. Several of these features were identified in our study group. The prevalence of oral/nasal involvement in the HIV-positive patients in our group was comparatively high (45 %), similar to findings reported by others [5, 13, 15]. In addition, EBV infection was also common (70 %) in our study group, with the highest relative incidence being in the PBL-HIV group as noted previously [5, 13]. However, our study group differed from other cohorts [13] in terms of having a comparatively low (20/50; 40 %) prevalence of HIV-positive patients.

There is no consensus yet regarding the optimal therapeutic approach for patients with PBL. Combination chemotherapy is commonly used, in addition to cART for HIV-positive patients. The current National Comprehensive Cancer Network guidelines recommend using intensive chemotherapy regimens such as CODOX-M/IVAC (cyclophosphamide, vincristine, doxorubicin, high-dose methotrexate alternating with ifosfamide, etoposide, and high-dose cytarabine), dose-adjusted EPOCH, or hyper-CVAD [16]. This is based primarily on data derived from case reports and small case series. In addition, regimens similar to those used to treat plasma cell myeloma that incorporate bortezomib and consolidation with high-dose chemotherapy followed by autologous stem cell transplant have been suggested [17]. Castillo et al. recommended recently frontline EPOCH (+/− bortezomib) with intrathecal prophylaxis during each cycle of EPOCH followed by consolidative high-dose chemotherapy and autologous SCT in first remission, if possible [13]. In our study, intensive chemotherapy showed similar or even worse results due to treatment-related complications compared with CHOP, in line with results identified in other studies [15, 18]. In addition, there was no apparent benefit of hyper-CVAD-based therapy over other regimens on EFS and OS (*p* = 0.186, *p* = 0.404). On the other hand, the number of PBL patients in our study group who received autologous SCT is too small to draw any conclusions.

The outcome of PBL patients in this study was similar to that reported by others, with a median OS of 6.5 months [5, 19]. Favorable prognostic factors documented in the literature include low stage, achieving clinical remission with chemotherapy, age <60 years, oral location, and absence of *MYC*/*IGH* gene rearrangement [12]. Our univariate analysis showed that age <60 years and low stage were associated with better OS. Although the use of cART in HIV-positive patients has been reported to improve outcomes among PBL patients [15], our findings and those of others have not been able to confirm the prognostic value of cART [20]. Additionally, in contrast to previous reports, we were unable to confirm the detrimental effect of *MYC* gene rearrangements in our patient cohort [5, 13, 20].

Immunophenotyping plays an important role in establishing a diagnosis of PBL. The neoplastic cells are commonly positive for CD138, MUM1, and CD38, and they are negative for CD45, CD20, and PAX5. Cytoplasmic immunoglobulins are expressed commonly [1]. However, the immunophenotypic profile of PBL is variable. Four cases in our series were negative for CD138. In these cases, the diagnosis of PBL was supported by additional markers such as MUM1, monotypic cytoplasmic immunoglobulin light chain expression, and EBER positivity combined with negativity for CD20 and PAX5. Additional markers including PRDM1/BLIPM1 and XBP1s have also been shown to be helpful in identifying a plasmablastic immunophenotype [21]. As described by others [14], CD10 expression (typically absent in normal plasma cells) was detected in a sizeable subset of cases (13/32; 41 %) in our study group.

It is essential to exclude other neoplasms whose clinical, morphologic, or immunophenotypic features might overlap with PBL. Such differential diagnostic considerations include anaplastic (or pleomorphic) plasma cell myeloma, extramedullary plasmacytoma (anaplastic, plasmablastic, and plasmacytic), DLBCL with plasmacytoid differentiation, anaplastic lymphoma kinase (ALK)-positive large B cell lymphoma, extracavitary or solid variant of primary effusion lymphoma (PEL), DLBCL associated with HHV8-positive multicentric Castleman disease, and EBV-positive plasmacytoma in immunocompetent patients (EPIC). Patients with plasma cell myeloma usually have elevated serum and urine paraprotein, lytic bone lesions, and other evidence of end-organ damage, and their neoplastic cells are rarely positive for EBV. Eight PBL patients in this series had bone marrow involvement in addition to systemic disease. All those tested were negative for serum paraprotein. It is noteworthy that there is no consensus in the literature regarding the utility of serum or urine paraprotein in distinguishing PBL from plasma cell myeloma, and some reported patients with PBL had an M-protein [14]. Another important entity in the differential diagnosis of PBL is DLBCL with plasmacytoid differentiation wherein the absence of immunosuppression and lack of EBV infection are much more common than in PBL. ALK expression defines a form of DLBCL that often can exhibit plasmablastic features. The presence of ALK expression by immunohistochemistry and the identification of *ALK* gene rearrangement typically establish the diagnosis of ALK-positive large B cell lymphoma. Extracavitary/solid variant of PEL can closely resemble PBL, but these tumors by definition are positive for the HHV8 virus [22–26]. DLBCL arising in HHV8-associated multicentric Castleman disease is similarly positive for HHV8 by definition [27]. The distinction between plasmablastic and anaplastic plasmacytoma and PBL is challenging. Similar to PBL, extramedullary plasmacytoma in general also frequently involves the head-and-neck region but, unlike PBL, only rare cases have been reported to be EBV-positive. The latter, recently termed EPIC, arise in the head-and-neck region or the gastrointestinal tract in immunocompetent patients [28]. EPIC lesions are composed of mature-appearing plasma cells. Unlike PBL, they lack a “starry-sky” pattern or cytologic atypia and often have a brisk CD8-positive cytotoxic T cell infiltrate in the background.

In summary, PBL is a rare neoplasm with variable clinical presentation and pathologic characteristics. Our study shows a higher frequency of primary extranodal involvement, HIV-negative status, and response to CHOP chemotherapy than has been commonly underscored in the literature. Our findings suggest that younger patients with low-stage disease treated with chemotherapy may have a favorable prognosis.

## Methods

### Patient group

We retrospectively identified patients diagnosed with PBL at our institutions (UTMDACC and NUS) between 1994 and 2013. Most cases were submitted in consultation or referred to our institutions after a biopsy was performed. All biopsy specimen slides were reviewed as a part of this study. Relevant clinical data including age at diagnosis, sex, HIV status, medical history, disease sites, Ann Arbor stage, therapies, and clinical outcomes were obtained from medical records. Patients with a history of plasma cell myeloma (PCM) were excluded from this study. This study was approved by the Institutional Review Board of The University of Texas M.D. Anderson Cancer Center in accordance with the Declaration of Helsinki.

#### Immunohistochemistry and in situ hybridization

Immunohistochemical studies were performed on formalin-fixed, paraffin-embedded tissue sections using standard methods. The antibodies and dilutions used were as follows: CD3 (1:100), CD19 (1:100), CD20 (1:1400), CD43 (1:100), CD45 LCA (1:300), CD79a (1:50), CD117 (1:100), CD138 (1:600), MUM1 (1:35), kappa (1:20,000), lambda (1:20,000), and Ki-67 (1:100) (Dako, Carpinteria, CA); CD4 (1:80), CD10 (1:50), CD38 (1:75), EMA (1:600), BCL6 (1:40), and HHV8 (1:50) (Leica Microsystems, Buffalo Grove, IL); CD5 (1:20) and cyclin D1 (1:40) (Thermo Fisher, Fremont, CA); CD56 (1:100) (Life Technologies, Grand Island, NY); CD30 (1:80) (Covance, Emeryville, CA); MYC (1:50) (Ventana Medical Systems, Tucson, AZ); and PAX5 (1:35) (BD Bioscience, San Jose, CA).

*In situ* hybridization (ISH) for EBV-encoded small RNA (EBER) was performed using a fluorescein-labeled peptide nucleic acid probe (Dako) in conjunction with the Dako peptide nucleic acid ISH detection kit for formalin-fixed, paraffin-embedded tissue sections.

#### Karyotyping and fluorescence in situ hybridization

Conventional G-band karyotype analysis was performed using standard methods as described previously [29, 30]. Karyotypes were reported according to the 2013 International System for Human Cytogenetic Nomenclature [31]. Fluorescence in situ hybridization (FISH) was performed on formalin-fixed, paraffin-embedded tissue sections as described previously [32]. Assessment for *MYC* rearrangement was performed using LSI *MYC* dual-color break-apart probe (Abbot/Vysis, Downers Grove, IL, USA), following the manufacturer’s instructions. Signals were analyzed using a fluorescent microscope (Carl Zeiss, Thornwood, NY). The cutoff for *MYC* gene rearrangement in our laboratory is >3.8 % nuclei with positive (break-apart) signals; however, all positive PBL cases in this study had numerous cells with positive signals, well above the cutoff level.

#### Statistical analysis

Survival was estimated by the Kaplan-Meier method. Overall survival (OS) was calculated from the date of diagnosis until death from any cause or last follow-up date. Event-free survival (EFS) was calculated from the date of diagnosis to the first event (disease progression or relapse). Survival curves were compared by the log-rank test. Differences between groups were considered significant if *P* values were less than 0.05 in two-tailed test. Multivariate analysis was performed by Cox proportional regression model to examine the relationship between survival time and patient characteristics.

#### Key message

Plasmablastic lymphoma is a rare neoplasm with lymphoid and plasmacytic differentiation that arises commonly, but not exclusively, in immunocompromised patients. HIV status has no impact on overall survival. The most significant prognostic parameters include age, stage, and EBV status. MYC deregulation is very common in PBL.

## Additional file

Additional file 1: Table S1. Karyotypic abnormalities identified by conventional cytogenetics in plasmablastic lymphoma cases. **Table S2.** Cox regression analysis results.

## Abbreviations

AD: Autoimmune disease
AIDS: Acquired immunodeficiency syndrome
ALK: Anaplastic lymphoma kinase
cART: Combined antiretroviral therapy
CLL/SLL: Chronic lymphocytic leukemia/small lymphocytic lymphoma
CODOX-M/IVAC: Cyclophosphamide, vincristine, doxorubicin, high-dose methotrexate alternating with ifosfamide, etoposide, and high-dose cytarabine
CHOP: Cyclophosphamide, doxorubicin, vincristine, prednisone
CVP: Cyclophosphamide, vincristine, and prednisone
DLBCL: Diffuse large B cell lymphoma
EBER: Epstein-Barr virus-encoded small RNA
EBV: Epstein-Barr virus
EFS: Event-free survival
EPOCH: Etoposide, vincristine, doxorubicin, cyclophosphamide, and prednisone
EPIC: EBV+ plasmacytoma in immunocompetent patients
HIV: Human immunodeficiency virus
hyper-CVAD: Hyperfractionated cyclophosphamide, vincristine, doxorubicin, and dexamethasone
IC: Immunocompetent
IgH: Immunoglobulin heavy chain
ISH: In situ hybridization
FISH: Fluorescence in situ hybridization
PBL: Plasmablastic lymphoma
PCM: Plasma cell myeloma
PEL: Primary effusion lymphoma
OS: Overall survival
PT: Post-transplant
