# Supplementary material for: Stage, age, and EBV status impact outcomes of plasmablastic lymphoma patients: a clinicopathologic analysis of 61 patients
Source: J Hematol Oncol. 2015 Jun 10;8:65. doi: 10.1186/s13045-015-0163-z (PMC4472407; doi:10.1186/s13045-015-0163-z)
Supplement: Additional file 1: Table S1. — Karyotypic abnormalities identified by conventional cytogenetics in plasmablastic lymphoma cases. Table S2. Cox regression analysis results. [file 13045_2015_163_MOESM1_ESM.docx]

**Supplemental Table 1.** Karyotypic abnormalities identified by conventional cytogenetics in plasmablastic lymphoma cases.

| **ID** | **Karyotype** |
| --- | --- |
| 4 | 43,Y,add(X)(p22.3),-8,add(8)(p21),-13,del(13)(q12q14),-15, add(17)(p11.2),-18,add(22)(p11.2),add(22)(p13)[17]  /46,XY[1] |
| 30 | 46,XY,del(6)(q23q29),t(8;14)(q24.1;q32),add(20)(p13)[10]/ 81~88,XXYY,del(6)(q23q29)x2,t(8;14)(q24;q32)x2,add(20)(p13)x2[cp9]/46,XY[1] |
| 38 | 47,XY,del(1)(q21q32),idic(1)(p13),del(5)(q15q33),del(6)(q21q25),del(8)(q23),add(14)(q32),add(16)(q24),+19,-20,der(21)t(9;21)(q13;p13),+mar[15]/46~47,XY,idem,add(6)(q25) [cp2]/ 46,XY[3] |
| 52 | 43,X,-Y,der(1)del(1)(p32p35)del(1)(q32),add(2)(p11.2),add(3)(q27),del(3)(q13.3),del(4)(q31.1q32),del(6)(q15q26),-7,-10,del(12)(p13),-13,-14,-17,+3~6mar[cp3] |
| 58 | 46,XY,t(8;14)(q24;q32),t(20;22)(q13.3;q13)[1]/46,idem,t(7;10)(q22;q22)[4]/46,XY[15] |

**Supplemental Table 2.** Cox regression analysis results

|  | *p*-value | HR | 95.0% CI for HR | |
| --- | --- | --- | --- | --- |
|  |  |  | Lower | Upper |
| Age at diagnosis | **.024** | 1.191 | 1.023 | 1.388 |
| Stage (high/low) | .086 | 8.450 | .738 | 96.726 |
| Bone marrow involvement | .964 | 1.063 | .074 | 15.239 |
| EBV status | .599 | 2.205 | .115 | 42.110 |
| HIV status | .200 | 28.105 | .172 | 4600.560 |
| “Immunocompetent” status | .857 | .733 | .025 | 21.555 |
